# Acute Intermittent Porphyria in an Adolescent Patient: Diagnostic and Treatment Challenges

**DOI:** 10.7759/cureus.74784

**Published:** 2024-11-29

**Authors:** Samuel B Rudnick, Nicole E West, Kelly Fong, Simon W Beaven, Nathan T VanderVeen

**Affiliations:** 1 Pediatrics, University of California Los Angeles, Los Angeles, USA; 2 Internal Medicine, University of California Los Angeles Vatche &amp; Tamar Manoukian Division of Digestive Diseases, Los Angeles, USA; 3 Internal Medicine, University of California Los Angeles David Geffen School of Medicine, Los Angeles, USA; 4 Internal Medicine, University of California Los Angeles Pfleger Liver Institute, Los Angeles, USA; 5 Internal Medicine-Pediatrics, University of California Los Angeles, Los Angeles, USA

**Keywords:** acute hepatic porphyria, acute intermittent porphyria, givosiran, hemin, hydroxymethylbilane synthase (hmbs), porphobilinogen, porphobilinogen deaminase, δ-aminolevulinic acid

## Abstract

Acute intermittent porphyria (AIP) is a rare inherited metabolic disorder caused by decreased activity of the enzyme porphobilinogen deaminase in the heme synthesis pathway. This leads to the accumulation of toxic porphyrin precursors, such as porphobilinogen and δ-aminolevulinic acid. Clinical manifestations typically include episodic bouts of severe neurovisceral pain and autonomic dysfunction. These events have various triggers, including fasting, dehydration, hormonal fluctuations, and certain medications, while treatment involves dextrose infusions for mild attacks and intravenous hemin for severe cases. This case highlights a 17-year-old female patient with numerous prior presentations to the healthcare system for intense bouts of abdominal pain, vomiting, and seizure-like activity who was ultimately diagnosed with AIP. Her course was complicated by diagnostic delays and challenges managing severe refractory pain requiring prolonged courses of hemin in addition to a multimodal pain plan. This case highlights the diagnostic complexities and treatment challenges that patients with AIP face when navigating this challenging clinical syndrome and identifies an opportunity for increased awareness to guide the testing and treatment for AIP.

## Introduction

Acute intermittent porphyria (AIP) is a rare inherited metabolic disorder characterized by deficient activity of the enzyme hydroxymethylbilane synthase (HMBS), leading to the accumulation of porphyrin precursors, porphobilinogen (PBG) and δ-aminolevulinic acid (d-ALA) in the blood and liver [1]. This disorder is a member of the porphyria family, which encompasses a collection of diseases caused by abnormalities in any of the enzymes involved in heme biosynthesis. AIP is one of the acute hepatic porphyrias, along with variegate porphyria and hereditary coproporphyria, which are characterized by periodic episodes of intense abdominal and limb pain in association with nausea, vomiting, and neuropsychiatric sequelae resulting from the accumulation of porphyrins in the liver and nervous system. Hematopoietic or cutaneous porphyrias, on the other hand, result from porphyrin-related bone marrow toxicity causing a photosensitive skin rash. Examples include porphyria cutanea tarda, erythropoietic protoporphyria, and congenital erythropoietic porphyria.

AIP has an estimated prevalence of five to ten cases per 100,000 individuals in Europe and North America [2]. While both White men and women are susceptible, women exhibit a higher prevalence of the disease and tend to experience more severe symptoms [3]. An estimated 83% of cases occur in females compared to males [4]. While cases of AIP have been identified in all major ethnic groups, the majority of reported cases have been in the non-Hispanic White population, with little clarity on a true ethnic predominance. Typically, AIP manifests in late adolescence or early adulthood, though cases across all age groups have been documented [5].

The clinical spectrum of AIP encompasses a myriad of symptoms, predominantly featuring acute neurovisceral attacks characterized by the classic triad of severe abdominal pain, neuropsychiatric manifestations, and autonomic dysfunction [4]. Intractable abdominal pain, often colicky and poorly localized, stands as the hallmark symptom, frequently accompanied by nausea, vomiting, and constipation [1]. Neuropsychiatric symptoms, such as anxiety, agitation, hallucinations, and seizures, may manifest during acute attacks [6].

Diagnosing AIP is done by first qualitative screening or quantitative measurement of urine porphobilinogen (PBG), δ-aminolevulinic acid (δ-ALA), and creatinine in samples protected from the light, as light may lead to rapid porphyrin decline and false negative results. The test processing time is over five days and it measures metabolites absorbed by ion exchange resins and quantified through ultraviolet-visible spectroscopy. Laboratory diagnosis is made by first establishing a substantial elevation of PBG of >10 mg/L or 10 mg/g of creatinine, though specific units reported may differ between laboratories. Treatment should be initiated in patients with a significantly elevated urine PBG or δ-ALA of more than 5x the upper limit of normal or positive qualitative screening. Genetic testing can be utilized for confirmation of the specific mutation causing the disease. Conversely, normal values during symptomatic periods confidently rule out the presence of acute hepatic porphyria. Stool and plasma testing is often also available for second-line testing, though not considered necessary for diagnosis [7].

The management of AIP requires a multifaceted approach aimed at both decreasing symptoms during attacks and preventing further acute episodes. Intravenous administration of hemin, a heme precursor, serves as the cornerstone of acute attack management, suppressing hepatic ALA synthase and mitigating ALA and PBG production [8]. Concurrently, supportive measures often require analgesia, antiemetics, and nutritional optimization for symptom alleviation [9]. Long-term management strategies revolve around trigger avoidance, including specific medications, alcohol, and fasting, with consideration for hormonal regulation in ovulating patients [10].

Despite predominantly affecting the nervous and gastrointestinal systems, untreated, severe, and prolonged AIP attacks can precipitate life-threatening complications. These may include progressive motor neuropathy leading to paralysis, respiratory failure secondary to diaphragmatic involvement, and cardiovascular instability (especially significant tachycardia and hypertension) due to autonomic dysfunction [11]. Furthermore, repeated attacks may incite chronic kidney disease as a result of recurrent hypovolemia and hypertension [2].

Diagnosing AIP is particularly challenging due to its vague symptomatology, often resulting in delays and misdiagnoses [5]. Managing AIP-related pain with opioid analgesics can lead to dependence, complicating treatment and prolonging recovery time. Moreover, demographic factors such as gender, race, ethnicity, and socioeconomic status may further contribute to delays in diagnosis, potentially worsening healthcare disparities and hindering prompt access to treatment [12,13].

AIP presents a complex clinical scenario characterized by a diverse clinical spectrum, diagnostic challenges, complex management strategies, and often prolonged rehabilitative course. Addressing these challenges necessitates heightened clinical suspicion, timely intervention, and a nuanced understanding of the demographic factors impacting disease presentation and management. This case report will highlight a rare instance of AIP in a pediatric patient, and discuss the diagnosis, management, and complications of her condition.

## Case presentation

A 17-year-old, previously healthy Hispanic female patient, presented to our hospital for a higher level of care with intractable abdominal pain of unknown etiology. She initially presented approximately one month earlier to an outside emergency department after a presyncopal event with subsequent lower abdominal pain, nausea, and emesis. She was diagnosed and treated for a urinary tract infection but was unable to obtain the full oral antibiotic course due to financial constraints. Three days later, she returned with worsening pain and emesis and was found to be tachycardic and hypertensive with leukocytosis to 21.9 K/µL. She was admitted to the hospital for management of presumed pyelonephritis and treated with ceftriaxone. She remained admitted for nine days, during which her leukocytosis peaked at 53.9 K/µL. She reportedly developed bloody diarrhea and underwent various modes of imaging including computed tomography (CT) of her abdomen and pelvis, which showed mild diffuse colitis and mesenteric lymphadenopathy. Stool polymerase chain reaction (PCR), presumably targeted at *Clostridium difficile *infection, was negative and she was treated with a seven-day antibiotic course. After reports of severe chest pain, she underwent CT chest and lower extremities and an echocardiogram, all of which were normal. Pain and leukocytosis improved, but she reportedly exhibited an episode of seizure-like activity, for which a CT head and electroencephalography (EEG) were performed and also normal; neurology consultants presumed this was psychogenic non-epileptic seizures (PNES). She was discharged home with no medications.

One week later, she returned again with severe lower abdominal and pelvic pain, nausea, vomiting, and anorexia. Laboratory findings were significant for severe acute kidney injury with creatinine of 2.9 mg/dL (ref. range: 0.5-0.9 mg/dL), increased from a baseline of 0.8 mg/dL. CT of her abdomen and pelvis was largely unremarkable. She was readmitted, and a nephrology consultant suspected that her acute kidney injury was a result of acute tubular necrosis from significant contrast exposure after numerous CT studies and nephrotoxic medications. Gynecology was consulted for pelvic pain with subsequently normal pelvic ultrasound. She experienced waves of severe abdominal pain, hypertensive urgency, tachycardia, and seizure-like activity which was minimally responsive to IV opioid analgesics. Abdominal radiographs remained normal. Three days after this admission, she was transferred to our facility.

On presentation, she was in severe distress due to abdominal, pelvic, and back pain, limiting her ability to engage in an interview. She was accompanied by her mother who helped relay additional information via Spanish video interpretation. On physical examination, she exhibited voluntary guarding with diffuse tenderness to palpation across her abdomen and thighs bilaterally. She expressed concern for decreased movement and sensation of her distal extremities, though she was able to ambulate independently, albeit painfully. Her vital signs were significant for persistent tachycardia of 150 beats per minute (bpm) and hypertension of 150/110 mmHg, even while asleep. Her EKG showed sinus tachycardia. For pain, IV acetaminophen and opioids were continued, though a multimodal pain regimen including topical analgesics, gabapentinoids, oxybutynin, and duloxetine failed to provide meaningful relief. For hypertension, hydralazine was used as needed. Gastroenterology was consulted, and an aggressive bowel regimen was started given concern for moderate stool burden on abdominal radiography. Further evaluation from gynecology, surgery, nephrology, and gastroenterology was unrevealing. Psychiatry was also consulted as diagnoses including somatic symptoms and factitious disorders were considered. A broad differential diagnosis was proposed and an initial laboratory workup was sent to reveal largely unremarkable complete blood counts, hepatic function panel, and markers of inflammation, with the results summarized in Table 1.

**Table 1:** Initial complete blood count (CBC), hepatic function panel, and inflammatory markers reveal largely unremarkable values, with mild leukocytosis with neutrophil predominance, normocytic anemia, and elevated ESR. AST, aspartate aminotransferase; ALT, alanine aminotransferase; ESR, erythrocyte sedimentation rate; CRP, C-reactive protein. µL, microliter; g, gram; dL, deciliter; fL, femtoliter; U, unit; L, liter; mm, millimeter; hr, hour; mg, milligram.

Procedure	Component	Value	Reference range
CBC and auto differential	White blood cell count	10.05	4.16-9.95x10^3^ / µL
	Absolute neutrophil count	8.55	1.80-6.90x10^3^ / µL
	Absolute lymphocyte count	0.86	1.30-3.40x10^3^ / µL
	Absolute eosinophil count	0.04	0.00-0.50x10^3^ / µL
	Absolute basophil count	0.03	0.00–0.10x10^3^ / µL
	Absolute monocyte count	0.50	0.20–0.80x10^3^ / µL
	Red blood cell count	4.30	3.96-5.09 x10^3^ / µL
	Hemoglobin	11.3	11.6-15.2 g/dL
	Hematocrit	35.0	34.9-45.2%
	Mean corpuscular volume	81.4	79.3-89.6 fL
	Platelet count	280	143-398x10^3^ / µL
Hepatic function panel	Albumin	4.0	3.9-5.0 g/dL
	Total protein	7.3	6.3-7.8 g/dL
	Bilirubin, total	0.6	< 0.8 mg/dL
	Bilirubin, direct	< 0.2	< 0.3 mg/dL
	Alkaline phosphatase	108	45-87 U/L
	AST	28	13-62 U/L
	ALT	19	8-70 U/L
Inflammatory markers	ESR	35	< 25 mm/hr
	CRP	< 0.3	< 0.8 mg/dL

Additional workup was sent for celiac disease, thyroid disorders, *Helicobacter pylori*, pheochromocytoma, and porphyria. Thyroid testing initially revealed subclinical hyperthyroidism with thyroid-stimulating hormone (TSH) 0.17 µU/mL (reference range: 0.3-4.7 µU/mL) and free T4 1.60 ng/dL (ref. range: 0.80-1.70 ng/dL), with subsequent normalization of TSH without intervention. Celiac testing was negative. Urine metanephrines were mildly elevated, though levels in the serum were normal and imaging of the adrenal glands was unremarkable, making pheochromocytoma unlikely. *Helicobacter pylori* stool antigen testing was positive, and the patient was started on quadruple therapy (bismuth, pantoprazole, clarithromycin, metronidazole) given local resistance patterns and her prior recent antibiotic course. Porphyria tests eventually resulted and are summarized in Table 2 below.

**Table 2:** Porphyria laboratory results reveal urine porphobilinogen levels >10x the upper-limit of normal as well as elevated uroporphyrin I, coproporphyrin I/II. ALA, aminolevulinic acid; CRT, creatinine. µumol, micromole; mol, mole; L, liter; µg, microgram; dL, deciliter; nmol, nanomole; sec, second.

Specimen	Test	Value	Reference range
Urine	Creatinine	494	1000-1800 mg/24 hr
	Porphobilinogen	120.0	0-8.8 µmol/L
Urine fractionated porphyrins	Uroporphyrin I	1039	0-4 µmol/mol CRT
	Coproporphyrin I	26	0-6 µmol/mol CRT
	Coproporphyrin III	21	0-14 µmol/mol CRT
Whole blood	Total porphyrins	86	<80 µg/dL
	Zinc-complexed protoporphyrin	76	<60 µg/dL
	ALA dehydratase	3.5	<3.5 nmol/L/sec
				Free protoporphyrin	11	<20 µg/dL
	Fecal	Fecal porphyrins	Negative	Negative

Given the laboratory findings displayed, hematology was consulted and a diagnosis of acute intermittent hepatic porphyria was made. Genetic analysis eventually confirmed a heterozygous mutation in the hydroxymethylbilane synthase (HMBS) gene associated with autosomal dominant AIP. Central vascular access was obtained, and she was started on intravenous hemin infusions at 3 mg/kg/day for a four-day-course, and later increased to 4 mg/kg/day (standard dosing) and extended to a 14-day course given suboptimal treatment response. Hydralazine was discontinued as it is known to potentially worsen symptoms of porphyria. She did exhibit improvement in symptoms of nausea with only mild improvement of abdominal pain despite normalization of her porphyria studies.

Her hospital course was complicated by a variety of factors. First, she developed significant opioid dependence due to medical management of severe abdominal pain during multiple visits to the emergency room and prolonged hospitalizations. She developed severe constipation leading to obstipation and megacolon, likely from a combination of her opioid regimen and the dysautonomia associated with AIP, negatively affecting her gastrointestinal motility. This required an aggressive bowel regimen and intermittent decompression with a rectal tube for gaseous distension. She experienced continued convulsive episodes and underwent an evaluation with neurology with a reassuring video EEG and brain MRI. She also developed moderate hyponatremia to 124 mEq/L secondary to a likely combination of syndrome of inappropriate antidiuretic hormone (SIADH), post-acute tubular necrosis diuresis, and known features of AIP. Additionally, her nutritional status remained poor despite dedicated oral and nasogastric tube trials, and she subsequently required several days of parenteral nutrition. 

Despite the completion of 14 days of hemin therapy, our patient continued to require high analgesic administration, including a hydromorphone patient-controlled analgesia (PCA) pump and ketamine. She experienced numerous presyncopal events as a result of the combined impact of porphyria, dehydration, and high-risk medications.

She was started on buprenorphine with assistance from our chronic pain subspecialists, ultimately facilitating her wean off the PCA pump. She was approved to start givosiran with standard dosing of 2.5 mg/kg injected subcutaneously once monthly. Givosiran is a small interfering ribonucleic acid (siRNA) targeting ALAS1 to reduce toxic metabolites, PBG and δ-ALA, in cases of refractory AIP. Once pain was adequately controlled, and porphyria flare was appropriately treated, the patient was safely discharged home with a plan to continue monthly givosiran injections and follow up with AIP specialists in the outpatient setting.

While the diagnostic urine porphyria studies were sent on the day of admission to our hospital, laboratory processing duration was seven days from admission and nearly one month from the initial presentation to the emergency room and subsequent outside hospital admission. She remained hospitalized for 64 days to manage the complications of her condition. A review of her medical record shows that she remains asymptomatic since discharge and continues with monthly givosiran injections, trigger avoidance, and laboratory screening as her maintenance therapy.

## Discussion

AIP stands as a notable challenge in both diagnosis and management among the inherited metabolic disorders. This discussion section seeks to address the many challenges associated with AIP, including diagnostic complexities, the propensity for opiate dependence in pain management, and compounding social risk factors exacerbating delays in diagnosis and care.

As noted in numerous other case studies, AIP presents a uniquely challenging diagnosis, perhaps most notably due to the vague presentation and low incidence [7]. While the triad of abdominal pain, neuropsychiatric symptoms, and autonomic dysfunction is classically seen, the range of presentations is highly variable and may include neuropsychiatric symptoms such as depression, anxiety, psychosis, phobias, agitation, delirium, and restlessness, as well as neurovisceral symptoms consisting of seizures, neuropathy, hyporeflexia, autonomic instability, abdominal pain, constipation, nausea, and vomiting [6]. However, the neuropsychiatric symptoms may overshadow other symptoms and make it less likely to consider AIP in the differential diagnosis. Additionally, the constellation of psychogenic non-epileptic seizures and pain paroxysms requiring opiate analgesics can potentiate biases in providers and lead to the perception of malingering. There are many potential triggers of an acute attack, including acute illness, fasting, medications, alcohol, and hormonal fluctuations. While not definitively known in this patient, triggers were likely multifactorial and included possible acute urinary and/or gastrointestinal infection, prolonged poor oral nutrition as a result of acute illness, and hormonal fluctuations related to ovulation.

With multiple organ system involvement, a comprehensive evaluation is required to reach a diagnosis. With this consideration, it is apparent why patients may experience significant delays in diagnosis, averaging eight to ten years from initial presentation and up to 15 years in some studies [5]. Patients may undergo unnecessary abdominal imaging and even surgeries such as appendectomy, cholecystectomy, or exploratory laparotomy prior to diagnosis. The literature also suggests many patients are misdiagnosed with acute surgical abdomen or gynecologic abnormality at some point during their illness course, as in our case [14]. Considering the severity of these painful attacks and the consequences of a delayed diagnosis, it is critical to highlight the diverse presentations of AIP so we may expedite the diagnosis and management of these cases to improve the lives and outcomes of these misunderstood patients. Providers must consider AIP in cases of unexplained, recurrent abdominal pain in people of child-bearing age and properly screen for the disease with measurements of urine PBG, d-ALA, and creatinine.

Unfortunately, clinicians and patients experience difficulty controlling symptoms even after a diagnosis is made and appropriate treatment is started. In fact, there is significant reported variability in response to treatment among cases. While hemin infusions are the most effective disease-targeted treatment, there is evidence of ineffectiveness or insufficient relief in over 25% of patients [5], as was the case with our patient. It remains unclear which factors may contribute to these refractory cases. There are no statistically significant associations between genetic mutations and symptoms, signs, or biochemical abnormalities in AIP. Though significantly less frequent with the implementation of safer prophylactic therapies, some refractory AIP cases ultimately require liver transplantation [15]. This point emphasizes the importance of investigating clinically accessible biomarkers or calculators to predict refractory cases to improve the care of these patients. 

There are limited evidence-based recommendations regarding pain assessment and management in AIP, despite acute pain being one of the most important clinical manifestations [16]. Opioid analgesics are ineffective at controlling pain in more than half of the cases studied [5]. Prolonged and high-dose opioid use can contribute to numerous dangerous side effects, as seen in our patient, such as gastrointestinal dysmotility, respiratory depression, dependence, and hyperalgesia. Thus, a possible explanation for poor recovery in this case may be iatrogenic as a result of a high opioid burden from a delay in diagnosis rather than refractory AIP.

We believe it is critical to highlight social factors that may have contributed to the delay in diagnosis in this case, further shedding light on important disparities in medicine. First, it has been shown in large-scale observational studies that women receive medical diagnoses longer after initial presentations than their male counterparts [17]. These trends persist despite controlling for the specific symptom or disease, and insurance coverage. This may be in part due to the fact that healthcare providers are historically trained to identify disease based on diagnostic criteria formulated from studies performed predominantly among White men, suggesting a deeply rooted disparity in medicine starting at the earliest levels of training [18]. Additionally, there is well-substantiated evidence suggesting differential management of symptoms such as abdominal pain depending on the race and gender identity of patients. For example, White patients are more likely than non-White patients, and males are more likely than females, to receive narcotics in the emergency department with abdominal pain across all pain scores [19]. This supports the notion that a non-White female patient presenting with acute abdominal pain may receive delayed care due to biases, whether implicit or intentional.

Additionally, while our patient was a fluent English speaker, she was accompanied by caretakers who spoke Spanish as their primary language. It has been well established that children of parents with limited English proficiency have worse health outcomes among healthy children. We suspect this holds true for sick or hospitalized patients as well, possibly due to suboptimal communication with providers, which is essential for clarifying details of the history of present illness, clarifying clinical uncertainty, and advocating for a child. In survey data, this population of patients and caregivers is less likely to report family-centered care [20].

We believe that the intersectionality of oppressions in this patient, including being an adolescent non-White woman with non-English-speaking parents, resulted in a cumulative negative impact that resulted in significant delays in her diagnosis, contributed to adverse outcomes, and ultimately, worse care of a vulnerable patient. Addressing these disparities through targeted training and increased awareness among healthcare providers is essential for improving the quality of care and outcomes for all AIP patients.

## Conclusions

AIP poses significant diagnostic and management challenges due to its diverse and often vague symptomatology, as illustrated by the case of our 17-year-old patient. Her case underscores the critical need for heightened clinical suspicion and comprehensive evaluation when encountering patients with unexplained neurovisceral symptoms. Despite the availability of targeted treatments, variability in patient response highlights the necessity for continued research to better understand and navigate refractory cases.

This case emphasizes the significant impact of social factors, including gender, ethnicity, and language barriers, on the timely diagnosis and management of AIP. The intersectionality of these factors can lead to biases and delays in care, contributing to worse outcomes for vulnerable patient populations. Improving outcomes for AIP patients requires raising clinical awareness, advancing research into effective treatments, and addressing systemic healthcare inequities. With this multifaceted approach, we can hope to provide timely and effective care to all AIP patients, mitigating the severe complications associated with this rare, debilitating disorder.
